# Supplementary material for: T cells discriminate between groups C1 and C2 HLA-C
Source: eLife. 2022 May 19;11:e75670. doi: 10.7554/eLife.75670 (PMC9177145; doi:10.7554/eLife.75670)
Supplement: Supplementary file 1. — Data for outer shell shown in parentheses. [file elife-75670-supp1.docx]

**Supplementary File 1.**

|  | **TCR9a-HLA-C*05:01-A18L complex** |
| --- | --- |
| **PDB code** | 7SU9 |
| **Data collection** |  |
| Temperature (K) | 100.00 |
| Space group | C 1 2 1 |
| *Cell dimensions* |  |
| *a*, *b*, *c* (Å) | 72.7, 74.2, 107.4 |
| *α*, *β*, *γ* (°) | 90.0, 101.3, 90 |
| Resolution range (Å) | 35.98 -1.993 (2.065 -1.993) |
| R _merge_ (%) | 9.9 (67.1) |
| *I*/*σ*(*I*) | 38.8 (2.8) |
| Completeness (%) | 98.5 (85) |
| Redundancy | 16.8 (6.4) |
| Total observations | 1267782 |
| Unique observations | 75490 (7166) |
| **Refinement** |  |
| Refinement resolution (Å) |  |
| *R*_work_ (%) | 17.9 |
| *R*_free_ (%) | 21.4 |
| *No. of atoms* | 7016 |
| Protein | 6510 |
| Water | 454 |
| Mean B-factor (Å^2^) | 39.78 |
| *rmsd from ideal values* |  |
| Bond lengths (Å) | 0.00 |
| Bond angles (°) | 0.69 |
| *Ramachandran statistics* |  |
| Favored | 97.13 |
