# Supplementary material for: T cells discriminate between groups C1 and C2 HLA-C
Source: eLife. 2022 May 19;11:e75670. doi: 10.7554/eLife.75670 (PMC9177145; doi:10.7554/eLife.75670)
Supplement: Supplementary file 2. [file elife-75670-supp2.docx]

**Supplementary File 2.**

| **TCR9a** |  | **Distance (Å)** | |
| --- | --- | --- | --- |
| **Alpha** | **HLA-C** | **C*08:02** | **C*05:01** |
| Asn33-ND2 | Arg62-NE | 3.9 | 3.4 |
| Asp34-OD1 | Arg62-NH2 | 3.0 | 3.0 |
| Asp95-OD2 | Arg62-NH2 | 3.0 | 3.5 |
| Asp97-OD2 | Lys66-NZ | 2.9 | 2.8 |
| Gln98-NE2 | Gln155-NE2 | 3.5 | 3.5 |
| Gln98-OE1 | Arg156-NH2 | 2.9 | 2.9 |
| Asn33-CA | THR163-CG2 | 4.0 | 3.9 |
| Met96-CE | THR163-OG1 | 3.4 | 3.6 |
| Thr32-CB | Glu166-CB | 3.7 | 3.8 |
| Tyr54-OH | Glu166-OE1 | 3.8 | 4.0 |
| Lys55-NZ | Glu166-OE2 | 3.7 | 3.0 |
| Thr32-CB | Trp167-CZ3 | 3.8 | 3.4 |
| Asn33-CB | Trp167-CZ3 | 3.9 | 3.9 |
| Thr32-OG1 | Arg170-CD | 3.4 | 3.4 |
| **Beta** | **HLA-C** | **C*08:02** | **C*05:01** |
| Glu97-OE1 | Arg69-NH2 | 2.3 | 2.4 |
| Asp99-OD1 | Arg151-NH1 | 3.1 | 2.9 |
| Val98-CG1 | Ala158-CB | 3.9 | 3.7 |
| Leu93-O | Gln155-NE2 | 3.2 | 3.1 |
| Arg97-NH2 | Ala158-CB | 3.8 | 3.9 |
| **Alpha** | **Peptide** | **C*08:02** | **C*05:01** |
| Gln98-O | Gly4-CA | 3.4 | 3.2 |
| Gln98-CD | Val5-O | 3.3 | 3.2 |
| Gln98-OE1 | Gly6-CA | 3.7 | 3.3 |
| **Beta** | **Peptide** | **C*08:02** | **C*05:01** |
| Tyr48-OH | Lys7-NZ | 3.8 | 3.2 |
| Glu49-OE2 | Lys7-NZ | 3.3 | 2.8 |
